# Use of machine learning to assess factors affecting progression, retention, and graduation in first-year health professions students in Qatar: a longitudinal study

**DOI:** 10.1186/s12909-023-04887-w

**Published:** 2023-11-30

**Authors:** Dalal Hammoudi Halat, Abdel-Salam G. Abdel-Salam, Ahmed Bensaid, Abderrezzaq Soltani, Lama Alsarraj, Roua Dalli, Ahmed Malki

**Affiliations:** 1 Academic Quality Department, QU Health, Qatar University, Doha, Qatar; 2 Department of Mathematics, Statistics, and Physics, College of Arts and Sciences, Qatar University, Doha, Qatar; 3 Student Data Management Department, Student Experience Department, Student Affairs, Qatar University, Doha, Qatar

**Keywords:** Health education, Student progression, Student retention, Student graduation, Machine learning, XGBoost

## Abstract

**Background:**

Across higher education, student retention, progression, and graduation are considered essential elements of students’ academic success. However, there is scarce literature analyzing these attributes across health professions education. The current study aims to explore rates of student retention, progression, and graduation across five colleges of the Health Cluster at Qatar University, and identify predictive factors.

**Methods:**

Secondary longitudinal data for students enrolled at the Health Cluster between 2015 and 2021 were subject to descriptive statistics to obtain retention, progression and graduation rates. The importance of student demographic and academic variables in predicting retention, progression, or graduation was determined by a predictive model using XGBoost, after preparation and feature engineering. A predictive model was constructed, in which weak decision tree models were combined to capture the relationships between the initial predictors and student outcomes. A feature importance score for each predictor was estimated; features that had higher scores were indicative of higher influence on student retention, progression, or graduation.

**Results:**

A total of 88% of the studied cohorts were female Qatari students. The rates of retention and progression across the studied period showed variable distribution, and the majority of students graduated from health colleges within a timeframe of 4–7 years. The first academic year performance, followed by high school GPA, were factors that respectively ranked first and second in importance in predicting retention, progression, and graduation of health majors students. The health college ranked third in importance affecting retention and graduation and fifth regarding progression. The remaining factors including nationality, gender, and whether students were enrolled in a common first year experience for all colleges, had lower predictive importance.

**Conclusions:**

Student retention, progression, and graduation at Qatar University Health Cluster is complex and multifactorial. First year performance and secondary education before college are important in predicting progress in health majors after the first year of university study. Efforts to increase retention, progression, and graduation rates should include academic advising, student support, engagement and communication. Machine learning-based predictive algorithms remain a useful tool that can be precisely leveraged to identify key variables affecting health professions students’ performance.

## Background

Across higher education, the persistence of university students until the completion of their educational degrees is a key indicator of student achievement, and therefore institutional success [1]. Student success parameters are commonly regarded as primary pointers of institutional performance, as they reflect the overall quality of student learning and intellectual involvement; integration of students in campus life; and effectiveness of programs in delivery of what students expect and need [2]. In this regard, student retention, progression, and graduation are considered essential attributes of students’ academic progress as well as institutional reputation [3]. Student retention refers to the ability of a higher education institution to keep the enrolled students engaged and motivated about their studies until completion of their degree, and it involves strategies to reduce attrition and increase the likelihood of students persisting in their education. Retention efforts may include academic support, mentoring, counseling, financial aid, and a supportive campus environment [4]. The majority of research on retention refers to this term as continued enrollment of a student from the first year to the second year [5, 6]. The importance of retention stems from the fact that higher retention rates at a given university indicate that more students will persist, pay tuition, and generate academic achievements; all of which are key factors to institutional achievement [7]. On the other hand, student progression refers to the advancement of students through various stages of their educational journey, with successful completion of coursework, attempting credits, and meeting specific academic requirements across different study levels. Student progression ensures that students make steady academic evolvement that culminates in earning their degrees in a timely and efficient manner [8]. Both retention and progression aim towards conclusion of student attainments that improve overall graduation rates [6, 9]. Higher education institutions keep records of retention, progression, and graduation rates of students as measures of program effectiveness [10, 11], as well as a requirement for accreditation [12, 13].

Several factors may affect retention and cause undergraduate students to leave their majors, such as low achievement in early years, competitive culture, specific atmosphere of some courses, curriculum overload, poor teaching quality, and loss of interest [14]. Research has highlighted methods to identify students at risk of dropping out and therefore low retention, such as analyzing college entry data, administering focused surveys, and implementing early warning systems [6]. Additionally, multiple factors affect student progression, such as students’ actual and perceived performance, enjoyment of the subject matter, personal contact with academics, student support services, and social attributes [15]. Across health professions education, factors such as gender and grades of first year were significant indicators of student dropout and progression in medical school [16]. Moreover, in a case study on retention of Doctor of Physical Therapy degree program, it was shown that implementation of a student support program based on advising, regular communication, expanded orientation, early academic warning, and campus services, among other features, was able to achieve better student success and increase graduation rates [17]. In nursing colleges, poor retention has been attributed not only to student academic factors, but also to a lack of necessary interventions by faculty starting from admission and persisting throughout the curriculum, with need for broad academic advising [18]. Despite such findings, a paucity of studies on factors affecting retention, progression, and graduation remains obvious in medical education literature, and this stresses the need for a thorough, long-term investigation as a part of quality assurance in health academia.

An emerging trend in higher education is to leverage empirical data using data mining techniques and machine learning strategies to analyze, predict and enhance student retention, progression, and graduation rates [19, 20]. One approach, which holds great promise in this field, involves the use of ensemble models that combine multiple algorithms of other base models to overcome the complexity involved in forecasting student retention and success [21–23]. Ensemble models, which include gradient boosting, random forest, and extreme gradient boosting, also called XGBoost, offer a powerful prediction solution that addresses the inherent limitations of single estimator models including low accuracy, high degree of variance, and bias. In comparison, ensemble models offer higher accuracy, lower variance and bias, improved predictive power, and higher interpretability [24]. XGBoost has demonstrated high performance, effectiveness, robustness, and accuracy in predicting student retention and success in higher education, as evidenced by benchmarked performance metrics when compared to other models [25]. One notable feature of XGBoost is its analytical capabilities in identifying key variables (feature importance analysis) that impact student retention, progression, and graduation, making it an invaluable evaluative tool in informing timely and strategic decisions and guiding interventions to enhance student retention and success [26]. The predictive power of XGBoost heavily relies on the selected variables during the modeling process. This underscores the importance of leveraging relevant expertise and crowdsourcing to engineer a rich set of variables that enhance the predictive accuracy [23]. XGBoost algorithm determines the relative importance of these different variables in predicting student retention, progression and graduation, thus helping in efficient allocation of resources [26]. It is crucial to recognize that the type and nature of variables play a significant role in deciding the predictive performance, effectiveness, and precision of XGBoost. Therefore, considering the institutional context becomes critical in defining the relevant variables, as there is no consensus on a universal model to apply uniformly across all higher education institutions. The customization and adaptation of the model to suit specific institutional needs and strategic priorities are critical for its successful implementation, and are appealing to examine in the context of health professions education.

Qatar University (QU) is the country’s primary institution of higher education, and has become today a beacon of academic and research excellence in the region. Since its inception in 1977, QU offers the widest range of academic programs tailored to meet the needs of Qatari society, with both national and foreign students enrolled. Subsequent to the approval of the Board of Regents in January 2017, QU established the Health Cluster, with three member colleges at that time, namely medicine, pharmacy, and health sciences. The Cluster currently includes two newer sister colleges, dental medicine and nursing. National and expat students from both genders are enrolled in the Health Cluster, and study together in a common first year established at the Cluster as introductory level of multi-professional education since 2018. The main concept behind this common year is to enhance the Health Cluster’s overall educational and financial effectiveness and to better utilize the existing resources in teaching, research, community service, and others. Accepted students are enrolled in the first common year before they are eligible to pursue their majors of selection among the five current health colleges. Once students successfully complete the first semester of this year, they are sorted and officially admitted into their selected colleges. It is worth mentioning that all health major students in the first year should take a block of common courses delivered through a team teaching approach by faculty from the different colleges of the Health Cluster. Although the offering and placement of these courses may vary according to the study plan of specific colleges, they remain regularly overseen by the Health Cluster. In this regard, the teaching material, theoretical and laboratory activities, and various assessments are delivered similarly to students from all health colleges. The effect of this unification of the common first year on health students’ success has not been assessed. Likewise, the study of factors affecting retention, progression, and graduation of students after the first year at the QU Health Cluster has not been previously realized, and to our knowledge, has not been done otherwise on health education majors in Qatar. This study aims to explore factors affecting health students’ retention, progression and graduation after finishing the first year of health education at QU Health Cluster, by XGBoost machine learning approach.

## Methods

### Study design

The study was a retrospective, longitudinal analysis of secondary data of students in the first year across the QU health cluster. Data for the study from the years 2015 through 2021 were extracted from the student data management system. The data were cured and analyzed to gain insights into factors affecting student retention, progression, and graduation.

### Data collection and types of data utilized

Secondary data of cohorts of students enrolled in the health colleges from 2015 until 2021 were collected from the QU data repository. A longitudinal design was chosen to collect all data over the specified timeline, enabling tracking changes and identifying patterns or trends. All students from the selected cohorts were included in the study rather than selecting a smaller sample from the target population to maximize the sample’s representativeness and reduce any potential bias that may result from selecting a subset of the data. Issues related to missing data, attrition, and other potential sources of bias that may arise over the studied years were carefully considered.

For each of the aforementioned cohorts, the data utilized included demographics of students like gender, nationality, college, high school type (whether public or private), high school Grade Point Average (GPA), and type of admission to QU (as QU permits direct admission from high school as well as admission to a preparatory phase called foundation year, where students receive English language and mathematics education). Moreover, data on student retention at QU, progression, and graduation were collected. For the purpose of analysis, student retention was defined as students who remained enrolled at QU after completion of the first year. On the other hand, student progression was defined as a change of student classification by earning more credit hours. For example, a student was considered progressed from freshmen to sophomore year if 29 credit hours have been earned within one academic year. If the classification of the student remained the same within one year, the student is not considered to have progressed. Finally, student graduation was referred to as completion of the degree program.

### Statistical analysis

Descriptive statistics were used to summarize and determine the cohorts’ characteristics and distribution of the students. The normally distributed data and results were reported with mean and standard deviation (SD), while the remaining results were reported with median and inter-quartile range (IQR). Categorical data were summarized using frequencies and proportions. The Chi-square (χ2) test or Fisher exact test assessed associations between two or more qualitative data variables. Quantitative data between the two independent groups were analyzed using the unpaired t-test or Mann-Whitney U test. Finally, univariate and multivariate linear regression analysis (controlling and adjusted for predictors such as age, gender, nationality, college, and others were applied to determine and assess the associations between academic performance and other attributes. The results of linear regression analysis were presented as coefficients with corresponding 95% confidence interval. All *P*-values presented were two-tailed, and *P*-values < 0.05 were considered statistically significant. All statistical analyses were performed using Statistical Package for Social Sciences (SPSS) software, version 28, and R program.

### XGBoost algorithm

XGBoost is a highly regarded machine learning algorithm that excels in both regression and classification problem-solving. Its optimized and distributed gradient-boosting framework offers exceptional efficiency, flexibility, and portability. In the current study, we deployed XGBoost to scrutinize the variables affecting student retention, progression, and graduation within our cohort of health major students.

The initial phase involved gathering an extensive dataset for the students. This dataset comprised demographic details (gender and nationality) and key academic performance indicators (student college, high school GPA, first achieved cumulative GPA, and whether a student had the first year of health major as common year). These factors were selected due to their potential impact on student outcomes. The target variable in the dataset was whether a student retained, progressed, or graduated within a designated timeframe.

The collected student data underwent a process of preparation and feature engineering, making it ready for analysis. Subsequently, we leveraged XGBoost to construct a predictive model. This algorithm builds a predictive ensemble model by iteratively combining weak decision tree models, a process grounded in the principle of boosting. Each successive decision tree in the ensemble is designed to rectify the inaccuracies of its predecessor. By examining the individual decision trees and their interplay, XGBoost captures the intricate relationships between the initial predictors and student outcomes.

During the model training phase, XGBoost monitors each feature’s usage frequency in pivotal decisions across all decision trees. The algorithm generates a ‘feature importance score’ for each predictor by aggregating these statistics. This score signifies the relative contribution of a feature to the model’s overall predictive strength. It is computed by tallying the total gain of each feature across all the decision trees in the ensemble, where ‘gain’ represents the enhancement in the model’s objective function achieved by bifurcating the data based on a specific feature. By scrutinizing the feature importance scores, the most impactful predictors in the model were identified. Features that bear higher scores were indicative of a more pronounced influence on student retention, progression, or graduation in health majors, hence understanding the critical factors that drive student outcomes. In our experiment, we used a grid search of parameters and cross-validation to ensure the best training and validation performance. We set also the parameter related to the class distribution. This parameter is used to manage the imbalance in classification problems where the class distribution is skewed. It is a form of scale factor applied to the positive class in binary classification, and its purpose is to give more emphasis to the minority class.

## Results

### Demographic characteristics of the cohorts

Table 1 shows the demographics of the studied group of students in terms of gender and nationality. Female Qatari students represent the majority of the studied population, with 88% of students. In terms of nationality, non-Qatari students represented 56% of the studied population, with about 12% higher proportion as compared to the Qatari students, and Chi-square showed a statistical association between gender and nationality with *p*-value < 0.05.

**Table 1:** Demographics of studied health professions student cohorts

**Nationality**	**Gender**	**Number (%)**
Qatari (43.9%)	Female	754 (88%)
	Male	99 (12%)
Non-Qatari (56.1%)	Female	876 (80%)
	Male	213 (20%)

### Student retention, associated factors, and characteristics of the non-returning students

**Fig. 1:**
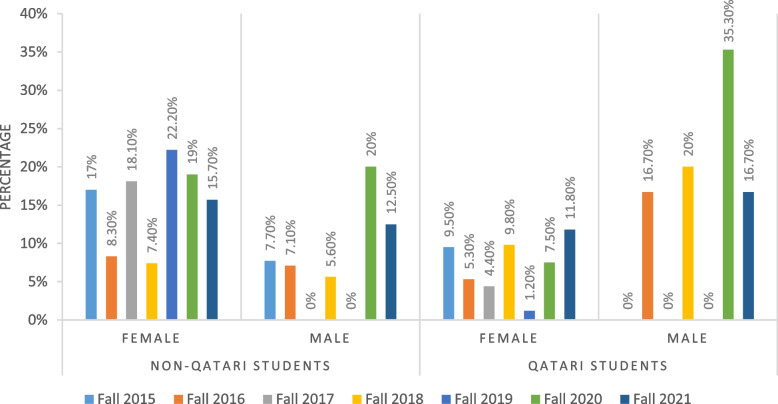
Percentages of non-returning health students in second fall after first year by nationality and gender

Figure 1 shows the proportions of non-returning health major students in the second fall semester after their first year by nationality and gender. All fall semesters had non-returning female students, with higher percentages for those who were non-Qatari. We noticed also that the percentage of non-returning male students was increased in the fall 2020 semester.

**Fig. 2:**
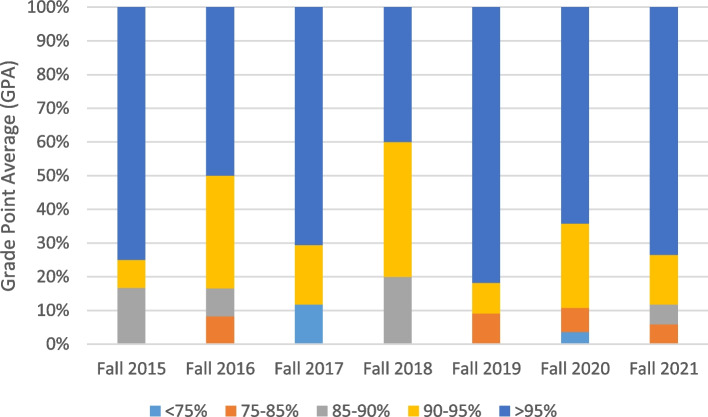
Percentages of non-returning health students in second fall after first year by high school GPA

Further breakdown by high school GPA category showed that the majority of non-returning students were highly competent upon initial admission to the QU Health Cluster, with high school GPA > 95 (Fig. 2).

We further analyzed the factors that affect students’ second fall retention. To achieve this goal, we trained an XGBoost classifier using student data that consists of demographic features and academic information. The Chi-square test showed no statistical association between the nationality and the second fall retention over time (*p*-value > 0.05). Eighty percent of the data was used to train the model, and 20% was used for testing. The XGBoost parameters were as follows: number of estimators = 300, learning rate = 0.05 and the maximum depth of trees = 7.The trained model achieved an accuracy of 83% on the test data. Table 2 details the ranking of features affecting student retention in terms of importance. The first achieved cumulative GPA during the first year and the high school GPA were the two most important predictors of the second fall retention, followed by student college. Less importance was given by the model to the demographic details of the students and common/non-common year feature.

**Table 2:** Ranking of features affecting health majors students’ retention in the second fall by importance

**Feature**	**Ranking**
First cumulative GPA	1
High school GPA	2
Student college	3
Nationality indicator (Qatari, Non-Qatari)	4
Gender	5
Common/non-Common year	6

Ranking assigns a value of 1 for highest importance and increasing numbers for decreasing importance

**Fig. 3:**
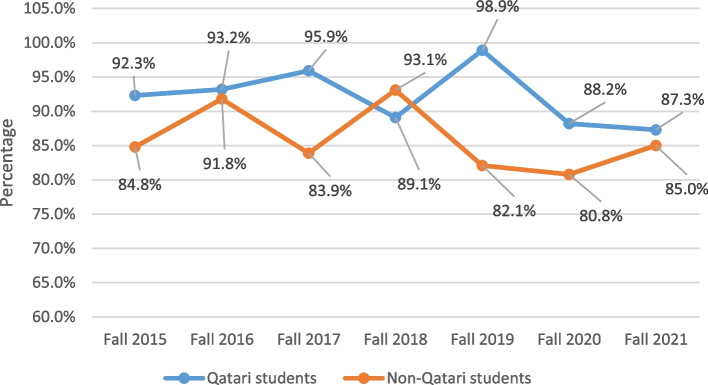
Trends of retention of health major students between fall 2015 and fall 2021 by nationality

**Fig. 4:**
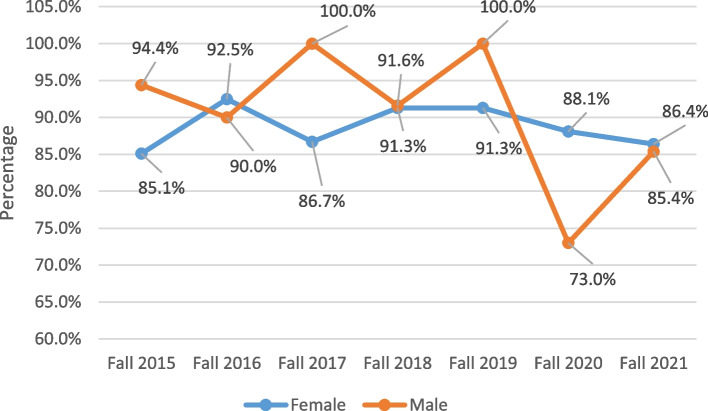
Trends of retention of health major students between fall 2015 and fall 2021 by gender

Further analysis showed that retention of health major students was in general highest in the college of medicine and lowest for students in the college of dental medicine. The fluctuation of retention rates across the years by nationality and gender were heterogeneous as shown in Figs. 3 and 4 respectively. However, both non-Qatari students (regardless of gender) and male students had the lowest retention rates in fall 2020, at around 81% and 73% respectively.

### Student progression and associated factors

We analyzed whether the change of the course level (first year courses versus major courses in the second study year) has an impact on student progression. Our strategy consisted of training a classifier on students’ data to determine whether a student will progress or not, then conduct a feature importance analysis which provides an estimate of the usefulness of each feature in the prediction. This analysis indicated the relative contribution of each feature to the model’s decision. For simplicity, the training data consisted of student demographics and an indicator of whether a student has progressed in the previous academic year (if applicable). We used an XGBoost classifier trained on 80% of the data with the following parameters: number of estimators = 100, learning rate = 0.01 and the maximum depth of trees = 5. The objective was to determine which of the indicators the classifier would heavily rely on when analyzing student progression. The findings, detailed in Table 3, showed that the progression in the previous year and the high school GPA were the two most important indicators, while less importance was given to nationality and student college. Analysis of data of progression by health college showed that it was highest for medicine and pharmacy students, and lowest for those in dental medicine.

**Table 3:** Ranking of features affecting health majors students’ progression by importance

**Feature**	**Ranking**
Progression in the previous academic year	1
High school GPA	2
How many times student progressed	3
Nationality indicator (Qatari, Non-Qatari)	4
Student college	5

Ranking assigns a value of 1 for highest importance and increasing numbers for decreasing importance

### Student graduation period and associated factors

**Fig. 5:**
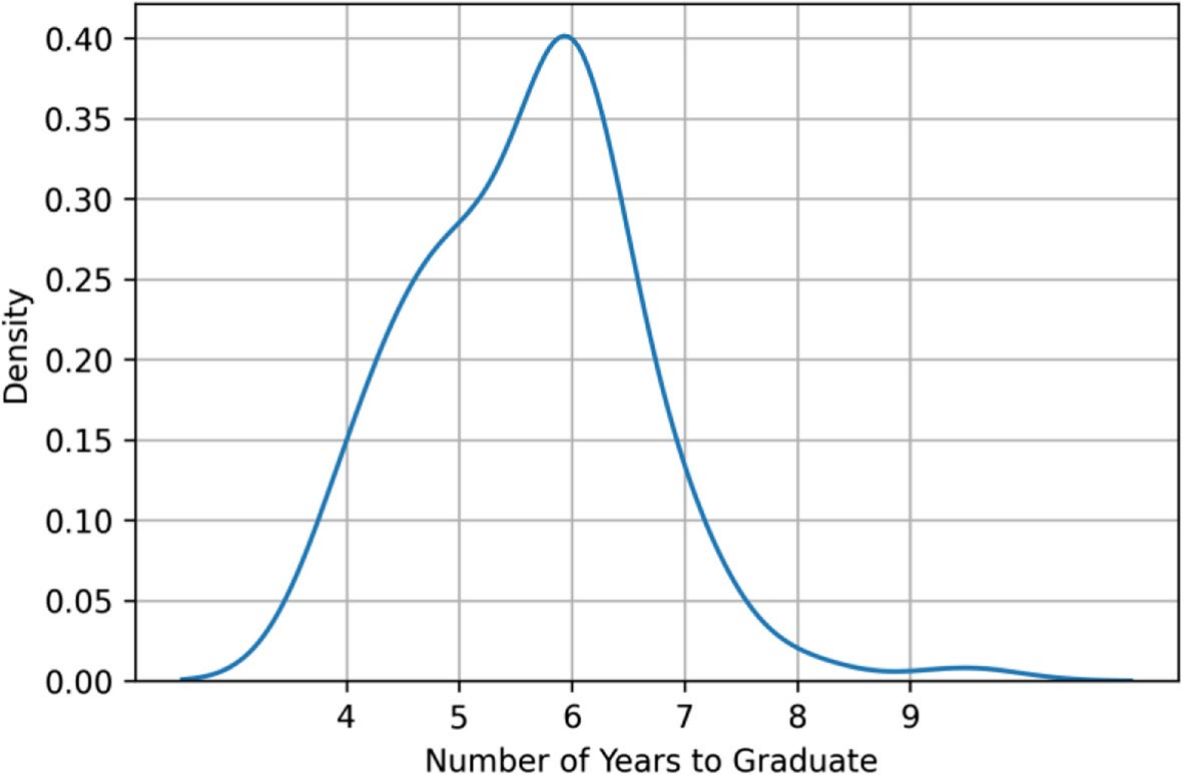
Density of the graduation period for the studied health major student cohorts

Analysis of the graduation period among the health student cohorts was done. Figure 5 depicts the density plot of the graduation period, where it was shown that most of the students managed to graduate from their health majors in 4 to 7 years.

**Fig. 6:**
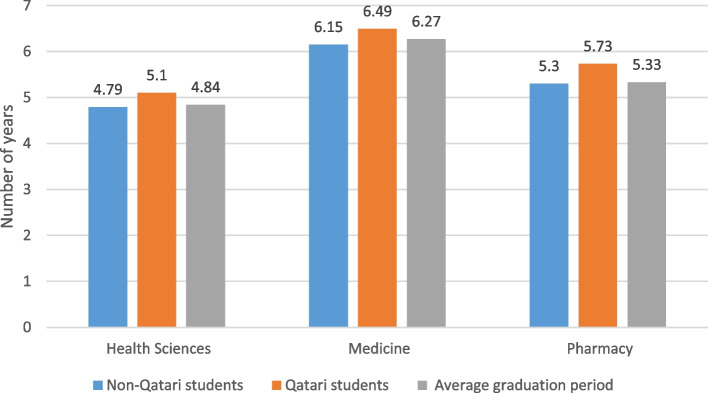
Mean graduation period in years by health college for the studied health major student cohorts

Further analysis of health majors students’ graduation period showed differences by college and nationality. Figure 6 details the average graduation period for the students as well as graduation period by health college and nationality (only three colleges are included, since the other two colleges did not graduate yet their first cohorts). The results show that the studied cohorts required slightly longer average period to graduate than the program length and that was highest for health sciences students (4.84 years for 4-year programs). Students in medicine and pharmacy majors needed slightly higher times to graduate than the program length in years (6 and 5 years respectively). In all majors, Qatari health students’ graduation periods were longer compared to non-nationals.

We also trained an XGBoost regressor to predict the graduation period using student data detailing their demographics and academic information. The model parameters were as follows: number of estimators = 200, learning rate = 0.02 and the maximum depth of trees = 5. Table 4 presents the feature importance, where the first cumulative GPA and high school GPA were highly important in predicting the graduation period. Less importance was attributed to the college, nationality, and retention in the second fall after admission to first year. However, common/non-common year indicator and gender were least important predictors of graduation.

**Table 4:** Ranking of features affecting health majors students’ graduation period by importance

**Feature**	**Ranking**
First cumulative GPA	1
High school GPA	2
Student college	3
Nationality indicator (Qatari, Non-Qatari)	4
Second fall retention (Yes/No)	5
Common/non-Common Year	6
Gender	7

Ranking assigns a value of 1 for highest importance and increasing numbers for decreasing importance

## Discussion

This study is among the few to address retention, progression, and graduation issues among students from different health professions education disciplines, and the first in Qatar to address this gap of knowledge. The collation of data of students majoring in different health professions and who are enrolled across several consecutive cohorts at QU is crucial for understanding factors associated with retention, progression, and graduation at a national level. This study contributes to providing general recommendations for improvement of the educational processes for various health major students and giving insights to assist them through successful completion of a health profession degree towards graduation.

In 2020, a systematic review on student success in health education majors suggested that it is challenging to isolate a single variable as the best predictor of student success; rather, a combination of variables can offer a reliable prediction method [27]. Hence, our model, based on machine learning, incorporated multiple elements in a comprehensive method of extracting intellectual insights from raw student data, that were analyzed to determine meaningful patterns of student performance in health professions education [28]. According to initial findings of this analysis, first academic year performance, followed by high school GPA, were common factors that respectively ranked first and second in importance in predicting retention, progression, and graduation. While student college ranked third in importance affecting retention and graduation and fifth regarding progression, the remaining factors showed a variable pattern of importance.

Retention analysis has become one of the most important attributes for higher education outcomes [29]. The loss of students returning to college for another year results in financial loss, less progressing students, and lower graduation rates for the institution. Also, this loss has negative consequences on stakeholders’, parents’, and students’ views [30]. In the studied health professions student cohorts, the first year GPA was the most important factor affecting retention, and this replicates previous findings in different universities [31, 32]. In health professions education, literature reports a similar pattern. For instance, in a large study from New Zealand, Shulruf and Colleagues [33] reported that first year grades are the most important determinant affecting first year success among students in nursing, pharmacy and health sciences undergraduate programs. Similar results regarding early academic achievement were reported by independent research groups for students from nursing baccalaureate major [34] as well as pharmacy programs [35]. Gershenfeld and Colleagues [36] showed that low first semester GPA is a statistically significant predictor of students’ inability to graduate on time. The cumulative GPA of the first year is a good indicator since it is a composite of average grades in all courses, and it will not only affect retention, but also progression and graduation rates according to our results. Therefore, institutions should determine best outcomes of the first year for their students and look into the competitiveness and challenges associated with this year. Special attention to students with low first year GPA should be practiced to improve retention of new comers to health majors and help them progress beyond their first year. This can be accomplished by various means including academic advising, strategies to promote overall student well-being, provision of resources for student support, and increasing student engagement. According to results shown in Fig. 1, most non-returning students were non-Qatari females; however, nationality and gender ranked fourth and fifth in importance regarding retention according to XGBoost predictive model. Meanwhile, compiling percentages of retention as shown in Figs. 3 and 4 by nationality and gender respectively showed a quite mixed pattern. It is noticeable that retention rates of male health major students and non-Qatari students were at minimum in fall 2020 across the studied cohorts. Although the direct interpretation of this pattern cannot be conclusively done from this analysis, it is anticipated that changes which accompanied COVID-19 at that time may have affected this cohort of students. For example, non-national students may have left the Health Cluster due to relocation of their families possibly caused by budget cuts and employment changes that occurred in the wake of the global pandemic. This effect of COVID-19 on student enrollment and retention rates is expected and has been previously reported in literature [37–39].

The high school GPA ranked second in importance for retention, progression, and graduation among the first year Health Cluster students. Similar results were shown by Stewart and Colleagues [40] and by Cambiano and Colleagues [41]. High school grades were also significant predictors of graduation in a 30-year retrospective study of students at medical school [42], and in another that included student data from a university for 6 consecutive years [43]. Contrary to our findings, Tross and Colleagues [44] reported that high school GPA had no predictive value for college retention. It is important to note that although high school GPA was the second most influential factor according to the predictive model, results reported in Fig. 2 show that the majority of non-returning students had high GPA in high school. This calls for a further analysis of these results and realizing an in-depth search of the factors that led these students to leave their health education. Literature provides several positive ways to improve retention like preadmission testing, cultural diversity, and faculty support of student success [45]. More specifically for health education programs, and according to results of a systematic review, the most successful strategies deployed by medical, nursing, and health colleges to improve retention were multi-layered. They consisted of appropriate selection to the program, comprehensive pre-entry orientation, supportive college environment, development of mentorship and tutoring programs, flexible content delivery, and providing social and financial resources [46].

Regarding health college of the studied cohorts, it ranked third in importance to affect retention and graduation, but last in affecting progression. We also observed that most students graduated within one year or less of the intended graduation period according to the individual programs, although Qatari students in general needed a slightly longer time. On the other hands, demographic factors like gender and nationality were consistently less important predictors according to our model. Also, the implementation of common year after 2018 as an early interprofessional education model was not an important driver of retention, progression, and retention. This needs further analysis perhaps after more cohorts of students who have enrolled in common year have graduated and the common year practices have been fully standardized. This is especially important in light of positive findings of interprofessional learning in the first year education among health students [47], and the pioneering experience of QU in this regard [48, 49].

Our study does have shortcomings. While the analysis relied on student demographic and academic data, it did not consider some non-academic factors that may affect the results. For example, student engagement, proper communication, and collaborative learning, are considered key components of a student success-oriented culture [17], and were not addressed in this analysis. Moreover, at some points during the period covered by the study, major challenges may have affected students, most importantly the COVID-19 pandemic and its profound effects on education in general as well as on health education [50–53], with universities struggling to maintain students and ensure reasonable retention rates [54, 55]. Changes in retention, progression, and graduation may have occurred as collateral effects of the pandemic, and were not possible to consider within this analysis. It is, therefore, crucial that more consolidated studies that are inclusive of additional factors affecting student success should be realized.

## Conclusion

To conclude, multiple factors affect retention, progression, and graduation rates for students at the Health Cluster at QU. This baseline analysis sheds a light on the importance on first year of university study and secondary education prior to college on student performance after admission to health majors. Improving rates of retention, progression, and graduation should consider a multitude of student factors, and should combine an approach of academic advising and student support to positively affect students’ progress in health professions education. Predictive methods rooted in machine learning, such as XGBoost, can be useful as an invaluable tool to automatically analyze complex student data with accurate results for uncovering important factors that influence health professions students’ accomplishments.

## Data Availability

The datasets generated and/or analyzed during the current study are available from the corresponding authors on reasonable request.

## References

[1] Kinzie J, Kuh G (2017). Reframing student success in college: advancing know-what and know-how. Change.

[2] Tinto V. Leaving college: rethinking the causes and cures of student attrition. Chicago: University of Chicago Press; 2012. 10.7208/chicago/9780226922461.001.0001.

[3] Hovdhaugen E, Frølich N, Aamodt PO (2013). Informing Institutional Management: institutional strategies and student retention. Eur J Educ.

[4] Burke A (2019). Student retention models in higher education: a literature review. Coll Univ.

[5] Cotton DR, Nash T, Kneale P (2017). Supporting the retention of non-traditional students in Higher Education using a resilience framework. Eur Educ Res J.

[6] Muller K, Feuer E, Nyman M, Sokolowski K, Rotella L (2017). Examining predictors of first year college student retention. N Y J Stud Aff.

[7] Gansemer-Topf AM, Downey J, Thompson K, Genschel U (2018). Did the recession impact student success? Relationships of finances, staffing and institutional type on retention. Res High Educ.

[8] Lloyd MG, Griffiths C (2008). A review of the methods of delivering HE programmes in an FE college and an evaluation of the impact this will have on learning outcomes and student progression. J Furth High Educ.

[9] Budden MC, Hsing Y, Budden CB, Hall M (2010). Heads or tails (success or failure)? Using logit modeling to predict student retention and progression. Contemp Issues Educ Res.

[10] Robertson S, Canary CW, Orr M, Herberg P, Rutledge DN (2010). Factors related to progression and graduation rates for RN-to-bachelor of science in nursing programs: searching for realistic benchmarks. J Prof Nurs.

[11] Elobaid M, Elobaid RM, Romdhani L, Yehya A (2023). Impact of the first-year seminar course on student GPA and retention rate across colleges in Qatar University. Int J Learn Teach Educ Res.

[12] Hardinger K, Garavalia L, Graham MR, Marken PA, Melchert RB, Nelson LA (2015). Enrollment management strategies in the professional pharmacy program: a focus on progression and retention. Curr Pharm Teach Learn.

[13] Kumar P, Shukla B, Passey D (2020). Impact of accreditation on quality and excellence of higher education institutions. Rev Invest Oper.

[14] Dagley M, Georgiopoulos M, Reece A, Young C (2016). Increasing retention and graduation rates through a STEM learning community. J Coll Stud Retent Res Theory Pract.

[15] Lowis M, Castley A (2008). Factors affecting student progression and achievement: prediction and intervention. A two-year study. Innov Educ Teach Int.

[16] Arulampalam W, Naylor R, Smith J (2004). Factors affecting the probability of first year medical student dropout in the UK: a logistic analysis for the intake cohorts of 1980–92. Med Educ.

[17] Noonan AC, Lundy M, Smith RA, Livingston BP (2012). A successful model for improving student retention in physical therapist education programs: a case report. J Phys Ther Educ.

[18] Mooring QE (2016). Recruitment, advising, and retention programs—challenges and solutions to the international problem of poor nursing student retention: a narrative literature review. Nurse Educ Today.

[19] Namoun A, Alshanqiti A (2020). Predicting student performance using data mining and learning analytics techniques: a systematic literature review. Appl Sci.

[20] Vehmas J, Khan AI, Kaliteevskii V, Chechurin L. Learning analytics overview: academic approach and machine learning possibilities. In: Digital teaching and learning in higher education: developing and disseminating skills for blended learning. Springer; 2022. p. 123–43.

[21] Imran M, Latif S, Mehmood D, Shah MS. Student academic performance prediction using supervised learning techniques. Int J Emerg Technol Learn. 2019;14(14):92–104.

[22] Niyogisubizo J, Liao L, Nziyumva E, Murwanashyaka E, Nshimyumukiza PC (2022). Predicting student’s dropout in university classes using two-layer ensemble machine learning approach: a novel stacked generalization. Comput Educ Artif Intell.

[23] Ornelas F. Estimation of persistence at a community college: a comparison of alternative machine learning models. Int J Comput Sci Eng Appl (IJCSEA). 2023;13(01). 10.5121/ijcsea.2023.13101.

[24] Kyriakides G, Margaritis KG. Hands-on ensemble learning with Python: build highly optimized ensemble machine learning models using scikit-learn and Keras. Packt Publishing Ltd; 2019.

[25] Attiya WM , Shams MB. Predicting Student Retention in Higher Education Using Data Mining Techniques: A Literature Review. Bangkok: International Conference On Cyber Management And Engineering (CyMaEn); 2023. p. 171–177. 10.1109/CyMaEn57228.2023.10051056.

[26] Yan K. Student Performance Prediction Using XGBoost Method from A Macro Perspective. Stanford: 2nd International Conference on Computing and Data Science (CDS); 2021. p. 453–459. 10.1109/CDS52072.2021.00084.

[27] Al-Alawi R, Oliver G, Donaldson JF (2020). Systematic review: predictors of students’ success in baccalaureate nursing programs. Nurse Educ Pract.

[28] Albreiki B, Zaki N, Alashwal H (2021). A systematic literature review of student’ performance prediction using machine learning techniques. Educ Sci.

[29] Jamelske E (2009). Measuring the impact of a university first-year experience program on student GPA and retention. High Educ.

[30] Lau LK. Institutional factors affecting student retention. Education. 2003;124(1):126–36.

[31] DeNicco J, Harrington P, Fogg N. Factors of one-year college retention in a public state college system. Res High Educ J. 2015;27:1–13.

[32] Raju D, Schumacker R (2015). Exploring student characteristics of retention that lead to graduation in higher education using data mining models. J Coll Stud Retent Res Theory Pract.

[33] Shulruf B, Li M, McKimm J, Smith M (2012). Breadth of knowledge vs. grades: what best predicts achievement in the first year of health sciences programmes?. J Educ Eval Health Prof.

[34] Newton SE, Smith LH, Moore G, Magnan M (2007). Predicting early academic achievement in a baccalaureate nursing program. J Prof Nurs.

[35] Allen DD, Bond C (2001). Prepharmacy predictors of success in pharmacy school: grade point averages, pharmacy college admissions test, communication abilities, and critical thinking skills. Pharmacotherapy.

[36] Gershenfeld S, Ward Hood D, Zhan M (2016). The role of first-semester GPA in predicting graduation rates of underrepresented students. J Coll Stud Retent Res Theory Pract.

[37] Howell J, Hurwitz M, Ma J, Pender M, Perfetto G, Wyatt J, et al. College enrollment and retention in the era of COVID. USA: College Board Publication; 2021.

[38] Willis K, Holmes B, Burwell N (2022). Improving graduate student recruitment, retention, and professional development during COVID-19. Am J Educ Res.

[39] Tanimowo RI, Tanimowo WO, Umeana MI, Tabeta BI (2022). Analyzing the effect of COVID-19 pandemic lockdown on students’ retention ability in selected science subjects. Univ J Educ Res.

[40] Stewart S, Lim DH, Kim J. Factors influencing college persistence for first-time students. J Dev Educ. 2015;38(3):12–20.

[41] Cambiano RL, Denny GS, De Vore JB (2000). College student retention at a midwestern university: a six-year study. J Coll Admiss.

[42] MaslovKruzicevic S, Barisic KJ, Banozic A, Esteban CD, Sapunar D, Puljak L (2012). Predictors of attrition and academic success of medical students: a 30-year retrospective study. PLoS One.

[43] Palacios CA, Reyes-Suárez JA, Bearzotti LA, Leiva V, Marchant C (2021). Knowledge discovery for higher education student retention based on data mining: machine learning algorithms and case study in Chile. Entropy.

[44] Tross SA, Harper JP, Osher LW, Kneidinger LM. Not just the usual cast of characteristics: using personality to predict college performance and retention. J Coll Stud Dev. 2000;41(3):323–34.

[45] Porter KB (2008). Current trends in student retention: a literature review. Teach Learn Nurs.

[46] Taylor EV, Lalovic A, Thompson SC (2019). Beyond enrolments: a systematic review exploring the factors affecting the retention of Aboriginal and Torres Strait Islander health students in the tertiary education system. Int J Equity Health.

[47] Imafuku R, Kataoka R, Ogura H, Suzuki H, Enokida M, Osakabe K (2018). What did first-year students experience during their interprofessional education? A qualitative analysis of e-portfolios. J Interprof Care.

[48] El-Awaisi A, Saffouh El Hajj M, Joseph S, Diack L (2016). Interprofessional education in the Arabic-speaking Middle East: perspectives of pharmacy academics. J Interprof Care.

[49] El-Awaisi A, Wilby KJ, Wilbur K, El Hajj MS, Awaisu A, Paravattil B (2017). A Middle Eastern journey of integrating Interprofessional Education into the healthcare curriculum: a SWOC analysis. BMC Med Educ.

[50] Aristovnik A, Keržič D, Ravšelj D, Tomaževič N, Umek L (2020). Impacts of the COVID-19 pandemic on life of higher education students: a global perspective. Sustainability.

[51] HammoudiHalat D, Safwan J, Akel M, Rahal M (2022). PROGRAMME DESCRIPTION: pharmacy education shift during times of pandemic and collapse: a perspective from a school of pharmacy in Lebanon. Pharm Educ.

[52] Hilburg R, Patel N, Ambruso S, Biewald MA, Farouk SS (2020). Medical education during the coronavirus disease-2019 pandemic: learning from a distance. Adv Chronic Kidney Dis.

[53] Ding A (2021). Medical education-collateral damage of COVID-19?. Postgrad Med J.

[54] El Said GR (2021). How did the COVID-19 pandemic affect higher education learning experience? An empirical investigation of learners’ academic performance at a university in a developing country. Adv Hum-Comput Interact.

[55] Urbina-Nájera AB, Cantón-Croda RM. Statistical analysis of university student retention strategies during the COVID-19 pandemic. Santos: IEEE World Engineering Education Conference (EDUNINE); 2022. p. 1-5. https://doi.org/10.1109/EDUNINE53672.2022.9782385.

